# A First Look at the Inhibitory Potential of *Urospatha sagittifolia* (Araceae) Ethanolic Extract for *Bothrops atrox* Snakebite Envenomation

**DOI:** 10.3390/toxins14070496

**Published:** 2022-07-17

**Authors:** Antonio L. Vera-Palacios, Juan D. Sacoto-Torres, Josselin A. Hernández-Altamirano, Andres Moreno, Maria C. Peñuela-Mora, David Salazar-Valenzuela, Noroska G. S. Mogollón, José R. Almeida

**Affiliations:** 1 Biomolecules Discovery Group, Universidad Regional Amazónica Ikiam, Km 8 Via Muyuna, Tena 150101, Ecuador; antonio.vera@est.ikiam.edu.ec (A.L.V.-P.); juan.sacoto@est.ikiam.edu.ec (J.D.S.-T.); josselin.hernandez@est.ikiam.edu.ec (J.A.H.-A.); gabriela.salazar@ikiam.edu.ec (N.G.S.M.); 2 Facultad de Ingeniería en Sistemas, Electrónica e Industrial, Universidad Técnica de Ambato, Ambato 180207, Ecuador; andyasmadres@gmail.com; 3 Grupo de Ecosistemas Tropicales y Cambio Global, Universidad Regional Amazónica Ikiam, Km 8 Via Muyuna, Tena 150101, Ecuador; mariacristina.penuela@ikiam.edu.ec; 4 Centro de Investigación de la Biodiversidad y Cambio Climático (BioCamb) e Ingeniería en Biodiversidad y Recursos Genéticos, Facultad de Ciencias de Medio Ambiente, Universidad Tecnológica Indoamérica, Quito 180103, Ecuador; davidsalazarv@gmail.com

**Keywords:** Amazon, antivenom, enzymes, plants, snakebite, toxins, Araceae

## Abstract

*Bothrops atrox* snakebites are a relevant problem in the Amazon basin. In this biodiverse region, the ethnomedicinal approach plays an important role as an alternative to antivenom therapy. *Urospatha sagittifolia* (Araceae) is a plant used for this purpose; however, its neutralizing properties have not been scientifically accessed. To fill this gap, we investigated the ability of *U. sagittifolia* to modulate the catalytic activity of *Bothrops atrox* venom, and their toxic consequences, such as local damage and lethality. The venom profile of *B. atrox* was assessed by chromatography and electrophoresis. Inhibition of the three main enzymatic and medically important toxins from the venom was evaluated using synthetic substrates and quantified by chromogenic activity assays. Additionally, the neutralization of lethality, hemorrhage and edema were investigated by in vivo assays. The possible interactions between venom proteins and plant molecules were visualized by polyacrylamide gel electrophoresis. Finally, the phytochemical constituents present in the ethanolic extract were determined by qualitative and quantitative analyses. The ethanolic extract reduced the activity of the three main enzymes of venom target, achieving ranges from 19% to 81% of inhibition. Our in vivo venom neuralizations assays showed a significant inhibition of edema (38.72%) and hemorrhage (42.90%). Additionally, lethality was remarkably counteracted. The highest extract ratio evaluated had a 75% survival rate. Our data support the biomedical value of *U. sagittifolia* as a source of natural enzyme inhibitors able to neutralize catalytically active *B. atrox* venom toxins and their toxic effects.

## 1. Introduction

Snakebite human envenoming is a complex medical challenge of the rural tropics, which largely affects indigenous communities [1]. In the Amazon, this burden on public health remains highly underestimated and with a high incidence [2]. The common lancehead pitviper, *Bothrops atrox* (Viperidae: Serpentes), plays a leading role on human envenomation in this region, being responsible for 70% of the cases registered annually [3]. The predominant clinical manifestations such as hemostatic disturbances and damaging tissue in these events are closely related to the proteomic composition of venom, which has a prevalence of phospholipase A_2_, metalloproteases and serinproteases [3,4,5,6]. The treatment of ophidian accidents is still dependent on a single centenary treatment based on animal heterologous immunoglobulins [7]. Despite the efficiency in neutralizing the systemic effects and the key contribution to reducing the number of deaths, antivenoms show some relevant limitations, such as adverse reactions, need for a specialized professional, cold chain, and batch variations. Some studies on Latin American antivenoms, including INS-COL (Colombia), PROBIOL (Colombia), ICP (Costa Rica), INS-PERU (Peru), UCV (Venezuela), BIOL (Argentina), FDTAV (Brazil), IVB (Brazil) and LBAV (Brazil) have demonstrated efficacy in neutralizing local effects under the preincubation protocol [8,9]. However, other investigations that addressed the preclinical efficacy of antivenoms via a challenge-then-treat model of envenoming have shown a low neutralization of these typical damages with significant improvements when a plant extract is used as a complementary therapy [10]. Generally, the poor neutralization of local effects is translated into clinical sequelae for the patient with social and economic impact [11]. Therefore, the search and development of innovative and complementary therapeutic modalities becomes a priority to decrease the impact of this neglected disease [12]. Taking these considerations into account, inhibitors targeting the abundant bothropic toxins represent an attractive possibility.

Plants are reservoirs of such biologically active compounds useful for the design of next-generation broad-spectrum therapeutic agents to complement the traditional therapy against snakebites [13]. Plant-derived toxin inhibitors with venom-neutralizing capacity may be advantageous over traditional therapy due to their accessibility, stability at room temperature, potential low cost, and ability to neutralize a wide spectrum of toxins, including those that produce rapid and destabilizing effects [14,15]. Particularly, Amazonian plants have shown a great potential as a chemical library of specialized metabolites able to modulate the enzymatic and toxic role of proteins and peptides from snake venoms [16]. Extracts of *Bellucia dichotoma* [17], *Connarus favosus* [18], *Philodendron megalophyllum* [19], *Dracontium dubium* [20], *Plathymenia reticulata* [18] and *Dracontium loretense* [21] are reported to inhibit some toxic activities such as edema, hemorrhage and lethality induced by *Bothrops* spp. venoms.

*Urospatha sagittifolia* (Rudge) Schott (Araceae) is a widely distributed plant species, found in the Amazon region of many countries, such as Bolivia, Colombia, Ecuador, French Guiana, Guyana, Paraguay, Peru, Suriname and Venezuela [22]. Ethnopharmacological reports from Guiana and Brazil have suggested its antivenom potential [23]; however, to date, these properties have not been scientifically validated. Traditionally, the tuber is prepared in a cataplasm with rum for treating snakebite and wounds of stingray [23]. Here, we evaluated the ability of *U. sagittifolia* (Rudge) Schott ethanolic extracts (USet) to interfere in the enzymatic and pharmacological activities induced by *B. atrox* toxins, using in vitro and in vivo assays.

## 2. Results

### 2.1. Biochemical Profile of B. atrox Venom

**Figure 1:**
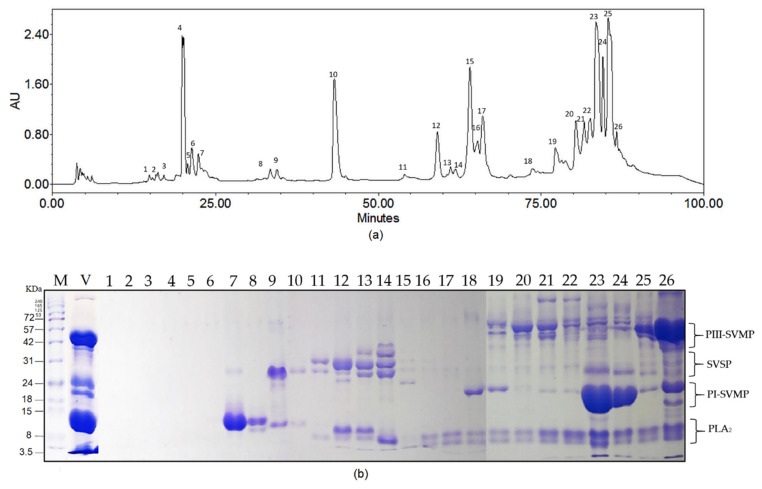
Biochemical profile of *B. atrox* venom pool (**a**) Chromatographic profile of the *B. atrox* venom pool set at 215 nm. (**b**) SDS-PAGE under reducing conditions shows the proteins contained in selected fractions. Molecular markers (M) (kDa) and crude venom (V) are shown on the left side of the gels followed by each of the numbered fractions (1–26).

The biochemical complexity of *B. atrox* venom assessed by RP-HPLC and electrophoresis revealed 26 major fractions (Figure 1). SDS-PAGE suggests the presence of phospholipases A_2_ (PLA_2_s, 10–14 kDa), lectins, PI metalloproteinase (PI-SVMPs, 18–25 kDa), serine protease (SVSPs, 30–42 kDa), PI metalloproteinase (PIII-SVMPs) and L-amino oxidases (LAAOs, 45–58 kDa). These *Bothrops* venom components have been found and characterized in previous studies [24]. The most evident chromatographic peaks correspond to the fractions eluted at 21, 43, 64, 84, 87, and 89 min, corresponding to 4, 10, 15, 23, 24, and 25. These findings suggested the predominance of PIII-SVMP y PI-SVMP in this pool.

### 2.2. Enzyme Inhibition Assays

**Figure 2:**
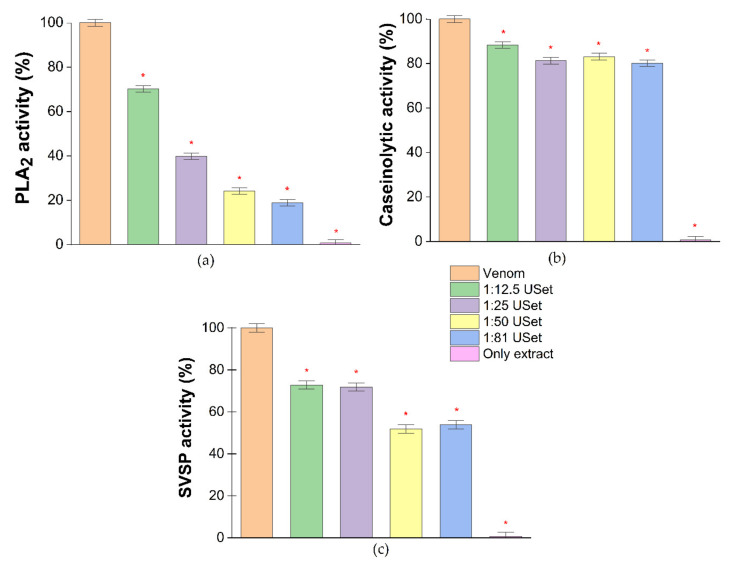
In vitro enzymatic inhibition of Uset treatments (**a**) PLA_2_; (**b**) caseinolytic; (**c**) serine protease activities of the *B. atrox* venom incubated with ethanolic extracts of *U. sagittifolia*. Results are expressed as percentages of enzyme activity, where crude venom is 100%. * = Statistically significant (*p* < 0.05) compared to venom.

*B. atrox* venom has considerable phospholipase, caseinolytic and proteolytic activity. These data corroborate the toxins observed in the gel. The enzymatic activity of bothropic toxins was significantly affected by the ethanolic extract of *U. sagittifolia* (USet). PLA_2_ activity was the most significantly inhibited, with reductions higher than 50% in three concentrations evaluated. The maximum inhibition of caseinolytic and proteolytic activity was 19.87% and 48.10%, respectively. The only plant extract sample did not show any enzymatic activity (Figure 2).

### 2.3. In Vivo Assays

#### 2.3.1. Edema

**Figure 3:**
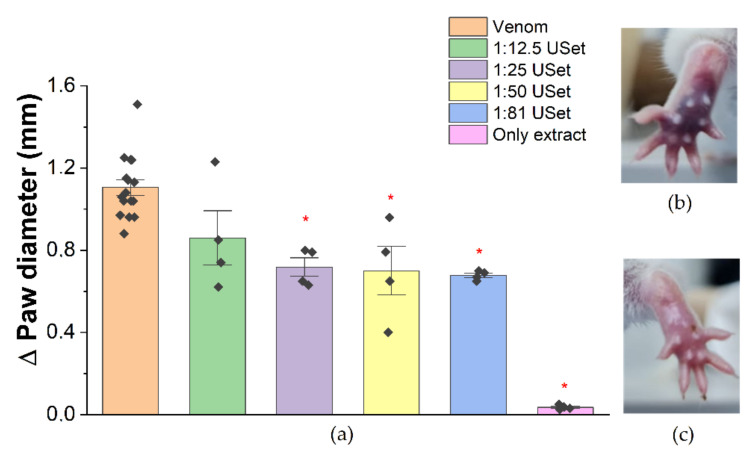
Inhibitory action of USet treatments on the edematogenic effect of *B. atrox* venom. (**a**) Differences in paw diameter. Paws were measured before and after (30 min) treatment injections. (**b**) Paw injected with crude venom (**c**) Paw injected with USet treatments. The difference in the diameter of the paw was used as an indicator of inflammation. * = Statistically significant (*p* < 0.05) compared to venom.

The neutralization of the inflammation by USet was recorded. There were significant edema reductions of 35.10%, 33.68%, and 38.72% by incubating venom:USet at 1:25/1:50/1:81 ratios, respectively (Figure 3a). In addition, evidence of simultaneous inflammation–hemorrhage inhibition was detected. There was a significant reduction in paw coloration, presumably induced by blood vessel rupture, when we compared crude venom (Figure 3b) with USet treatments (Figure 3c). Plant extract did not cause paw inflammation.

#### 2.3.2. Hemorrhage

**Figure 4:**
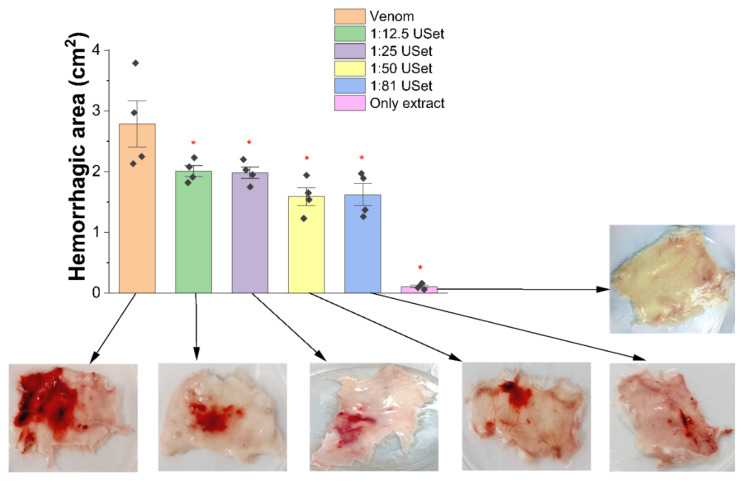
Inhibition of *B. atrox* venom-induced hemorrhage by *U. sagittifolia* extracts. Differences of hemorrhagic areas between crude venom and USet-preincubated solutions are shown. Representative photographs of each treatment studied. The results are expressed as the hemorrhagic areas (cm^2^). * = Statistically significant (*p* < 0.05) compared to venom.

The venom caused mobility problems, pain, and extensive hemorrhagic halos in the dorsal region of tested mice. Three hours after the injection, a significant reduction in hemorrhage was found for all USet treatments (Figure 4). Area reductions were 27.65%, 28.81%, 42.90%, and 41.71% for ratios 1:12.5/1:25/1:50/1:81, respectively. Hemorrhagic halos were not observed when only plant extract was injected. Treated mice skin pictures in Figure 4 show the reduction in hemorrhagic areas and dermal clots compared with the crude venom.

#### 2.3.3. Lethality

**Figure 5:**
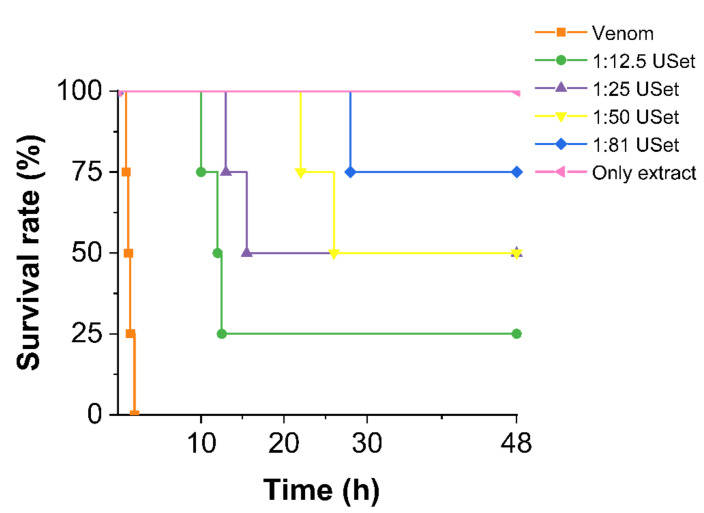
Survival curve of mice injected with venom and USet treatments. The extract improves the survival rate and, delays death. The lethality assay was recorded for 48 h.

The pool of *B. atrox* venom caused signs such as weakness, tachypnea, dizziness, slow movement, and sudden death in mice. Animals that received a 180 µg of crude venom died within 2 h. The ethanolic extract of *U. sagittifolia* seems to be effective to delay and prevent death at higher doses. We found survival rates of 25% (1:12.5), 50% (1:25/1:50) and 75% (1:81) obtained in 48 h (Figure 5).

The lethality of USet extract was evaluated at high doses (2000 µg/g). Behavioral changes and death in mice were not recorded. All the mice showed normal motor skills, surviving the 48 h trial. Thus, there is no evidence indicating that the extracts are deadly.

### 2.4. Venom–Extract Interaction

**Figure 6:**
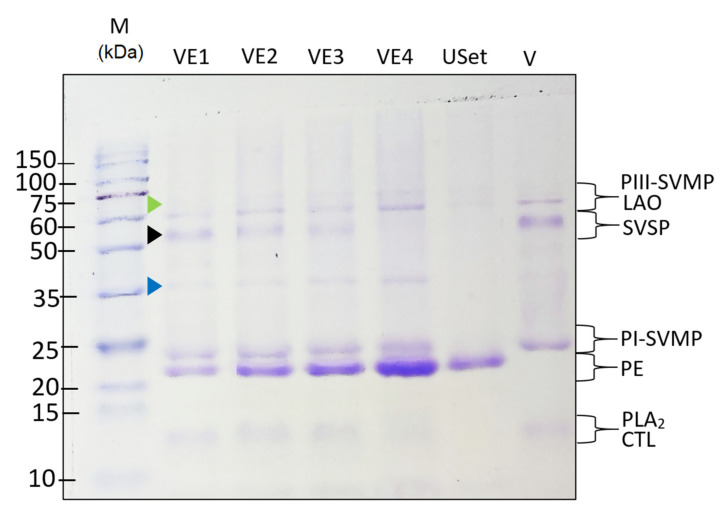
Interaction of venom–extract components in different USet ratios. SDS-PAGE under reducing conditions shows the proteins contained in the venom pool (V) and the venoms incubated with the extract. Molecular marker (M1) (kDa) is shown on the left of the gel. VE1 = 1:12.5 USet, VE2 = 1:25 USet, VE3 = 1:50 USet, VE4 = 1:81 USet, USet = Only ethanolic extract.

SDS-PAGE showed the crude venom (V) keeps the same components found in the biochemical profile. The electrophoresis evidenced that the extract is composed by proteins with a mass of 21–23 kDa, which were present in all treatments and increase its pigmentation with higher ratios. The venom proteins are conserved in all treatments, except in VE4, where the 50–56 kDa band (SVSP) disappears. This band loses intensity as the dose of extract is increased (black arrowhead). Interestingly, bands of unknown 35 kDa (blue arrowhead) and 72 KDa proteins (green arrowhead), which are not present in the crude venom (V) or extract (USet), increases their pigmentation as the SVSPs decrease (Figure 6). This suggests both, the precipitation of proteins and/or the formation of molecular complexes, which can be related to the inhibition of enzymatic activity.

### 2.5. A View on Phytochemical Profile of Ethanolic Extract from U. sagittifolia

The phytochemical screening evidenced the presence of saponins, terpenoids, tannins, phenolic, alkaloids, coumarins, and flavonoids. Our findings suggest that these four last compounds are abundant (Table 1). Consistent with this, the quantitative colorimetric analysis confirmed a significative content of polyphenols (5.97 ± 0.10 mg GA/g of extract) and flavonoids (2.20 ± 0.30 mg CT/g of extract).

## 3. Discussion

Disabilities induced by snake venom are highly frequent, being about approximately five times higher than the number of deaths caused by bites [1]. These local effects caused mainly by catalytically active toxins are poorly countered by currently available therapy. These lead to loss of limb function, which generate economic losses and social problems for vulnerable groups. In many neglected communities around Amazonia with weak health system, the use of plants and their extracts remains a commonly reported strategy due to delayed access to official treatment [25,26,27,28].

Alternative therapies for envenoming, such as enzyme inhibitors, have been tested in recent years due to their wide range of inhibitory activity and less secondary effects [7]. Exogenous and endogenous [29] peptides, small chemical inhibitors [30,31], and plant-derived metabolites are becoming trend strategies to fight snakebites [14]. Several potent natural components produced from plant species, such as *Aristolochia indica* [32] and *Azadirachta indica* extracts [33], have been shown to help reduce inflammation, local tissue damage, and acute pain produced by snakebites. Amazon plants such as *Philodendron megalophyllum* have demonstrated an inhibition of hemorrhagic and fibrinolytic activity [19]. Thus, plants represent a source of enzyme inhibitors, which can be alkaloids such as MMV (12-methoxy-4-methyl voachalotine) [34], acids (Aristolochic Acid) [34], steroids (β-sitosterol) [16], flavonoids (rutin) [34,35], coumestans (wedelolactone) [34], pterocarpans (Edunol) [13], terpenoids (Bredemeyeroside B) [36], and tannins (Ellagic acid) [37].

*U. sagittifolia* is a shallow-water emergent plant found in swamps, ponds, and stream banks, and it is the genus’ most extensively dispersed species, with populations distributed in the Amazon basin [38]. In countries such as French Guiana, Colombia, and Ecuador, ethnopharmacological studies have suggested the possible antivenom effect caused by plant tubers [23,39]. Macerated, boiled, or crushed tuber is often administered directly to the snakebite site [24]. Although ethnopharmacologically documented, there is no scientific research on its chemical constituents and pharmacological properties. Here, we discuss the neutralization of a wide variety of venom activities by *U. sagittifolia* (Rudge) Schott ethanolic extracts, which is a widely distributed species along the Amazon basin, usually found in shallow water swamps, ponds and stream banks [38].

The species of snake studied, *B. atrox*, together with *B. asper* and *B. jararaca* cause 70,000 bites per year throughout South and Central America and produce high numbers of morbidity and disabilities in those bitten [40]. The venom of *B. atrox*, like other bothropic venoms, is myotoxic, proteolytic, and hemorrhagic, producing local effects such as edema, inflammation, and necrosis [41]. Systemic responses by *Bothrops* envenoming such as shortened coagulation times, renal failure, bleeding, and even death, particularly if the treatment is not timely. The principal components of *B. atrox* responsible for the toxic and pharmacologic effects are SVSPs, which produce coagulopathy, SVMPs, which cause hemorrhage, and PLA_2_s, which causes edema and myotoxicity [42].

We initially accessed the biochemical profile of *B. atrox* venom in order to gain a molecular picture of its composition. The pool showed all hallmark components of this sample previously characterized by our group. The Ecuadorian *B. atrox* venom is primarily hemorrhagic, with high abundancy of PIII-SVMPs and PI-SVMPs [43]. These results are supported by research carried out in Colombia and Brazil [44] where metalloenzymes are the most abundant venom’s components, particularly PIII- SVMPs and PI-SVMPs. Thus, the pool employed in this study is a representative sample of *B. atrox* venoms found in this region.

The enzymatic activity of the venom was studied to define the neutralizing capacity of the extract. USet seems to inhibit the three enzyme families evaluated in a dose-dependent manner, achieving significant reductions in catalytic activity of 81.3%, 19.8%, and 48% for PLA_2_, SVMP, and SVSP, respectively. Similarly, enzyme inhibition has been reported for other extracts. For instance, the compound “AIPLAI” was isolated from the methanolic extract of the leaves of the plant *Azadirachta indica* inhibited venom PLA_2_ in a dose-dependent manner, with reductions in activity ranging from 10% to 60% [33]. Flavonoids from citrus fruit extracts, particularly hesperetin, inhibit two SVSPs expressed by *Crotalus simus* [45]. Macrolobin A, derived from *Pentaclethra macroloba* extracts, reduced PI-SVMP and PIII-SVMP-induced proteolytic activity by up to 90% and 80%, respectively [46].

Our enzymatic results support the findings obtained from in vivo essays, which show a considerable decrease in the venom’s toxic effects. In *Bothrops spp*. envenoming, edema is a common local effect with an increase in volume, immune cell migration, and tissue degeneration in the affected area [24]. Its rapid emergence challenges the antivenom neutralization. Mice paws showed these effects, with a considerable increase in the circumference of the paw and noticeable color changes. However, paws treated with *U. sagittifolia* extracts evidenced a significant decrease in venom-induced edema up to 39%. Other studies have highlighted the anti-inflammatory effects of plants extracts. Caro et al. found that ethanolic extracts of *D. dubium* have an edema-inhibitory action that is even better than antivenom of heterologous proteins tested with a decrease in the paw volume close to 46.7% [20]. Other plants, such as *Piper longum* [47], *Cordia verbenacea* [48], *Loasa speciosa* [49], *Chaptalia nutans* [49], and *Uncaria tomentosa* [49] (Table 2) also showed a statistically significant dose-dependent decrease in venom-induced edema.

Another typical local effect of *Bothrops* envenoming is hemorrhage. The biochemical profile of the pool showed high abundancy of pro-hemorrhagic enzymes that can produce tissue injury, blood loss, anemia, hypovolemic shock, and death. Mice receiving plant treatment had considerably fewer effects, including the inhibition of hemorrhagic activity (42.9% at high dosages). Previous research in Latin America showed that extracts of *D. dubium* reduces up to 95% of the hemorrhagic area induced by *B. asper* venom [20]. There are other herbal extracts that also considerably inhibit the hemorrhagic activity of bothropic toxins: *Mouriri pusa, Byrsonima crassa, Davilla elliptica, Strychnos pseudoquina* [50], *Connarus favosus* [51], *Bursera simaruba, Clusia torresii, Croton draco, Persea americana, Phoebe brenesii, Pimenta dioica, Sapindus saponaria, Smilax cucμlmeca* and *Virola koschnyi* [52] (Table 2). Despite hemorrhagic inhibition in animals, SVMP has less enzymatic activity inhibition when compared to PLA_2_ and SVSP. Because azocasein is a nonspecific substrate, the reduction in SVMP enzymatic activity cannot be linearly and directly connected to the suppression of hemorrhage [53].

On the other hand, systemic effects of envenoming, such as lethality, are caused by a complex interaction of toxins and the accumulation of mediators derived from tissue destruction [54]. The USet treatments in a dose-dependent manner resulted in a higher survival rate and extended the time of death. The neutralization of lethality appears to be caused by the suppression of PLA_2_, SVSP, and SVMP linked to the local and systemic effects of *Bothrops spp*. venoms. Even at large dosages, none of the USet ratios investigated reached a 100% survival. Interestingly, *D. dubium* extracts have shown to completely prevent the death of animals [20] and to significantly boost the survival rate when compared to the control. Plants such as *Piper longum* exhibited results (survival rate between 50–100%) [47] similar to the findings of this investigation.

Other American plants that are not phylogenetically related to *U. sagittifolia* have shown 100% lethality neutralization, such as *Harpalyce brasiliana* in northeastern Brazil and the Mexican herb *Brongniartia podalyrioides*, where edunol, a pterocarp derived from the plant root, neutralizes the lethal effects of crude *B. jararacussu* and *B. atrox* venoms (Table 2) [13,34]. Plants such as *Piper longum* exhibited results (survival rate between 50–100%) [47] similar to the findings of this investigation. Since the neutralization caused by natural molecules found in plants is not linked to antibodies but associated with broad-spectrum inhibition mechanisms such as blocking enzymatic active sites or ion chelation [55], it is likely that *U. sagittifolia* ethanolic extract contains antivenom activity for other venomous snake species, as other plant extracts have shown [56,57].

Different approaches have been employed to assess the pharmacological potential of plants in venom research. Pre-incubation is the main and traditional approach, but recently, other strategies have been emerging. De Moura et al.’s [58] study is an illustration of this point. These authors evaluated the pre-treatment and post-treatment, where the extract of *Bellucia dichotoma* was applied before and after the injection of *B. atrox* venom, respectively. In summary, their findings showed important differences, which challenges the clinical translation of pre-incubation results. This point raises crucial questions about plant extract efficiencies in various conditions and should be considered in future works. Additionally, research has been performed on the synergy of heterologous protein and plant extracts. In this scenario, Stuani et al. [59] found that combining both antivenom and plant extracts for neutralizing *Crotalus durissus* venom results in a statistically significant rapid recovery from toxic effects in animals compared to the *Mikania glomerata* extract only group.

SDS-PAGE electrophoresis was used to assess if the proteins formed molecular complexes or were precipitated after being exposed to the USet treatments. Except in the sample with the greatest ratio (VE4), the venom protein bands were preserved in all treatments. The conservation of the venom bands in the samples incubated with USet means that the extract inhibits the enzymes, not degrades them. However, as the USet ratio increases, the band associated with SVSP (≈60 KDa) loses pigmentation, and two bands at 72 and 35 kDa appears. The presence of these bands with a larger molecular weight in the USet treatments possibly indicates that the venom–extract interaction has promoted the creation of protein complexes. Additionally, the largest ratios of USet inhibits serine proteinases, probably by inducing structural degradation that caused the formation of proteins with lower molecular. Another reasonable explanation for this phenomenon is venom protein precipitation, as documented by Vale et al. [57], in which high amounts of *Schizolobium parahyba* plant extract cause protein charge instability. In addition, there is a band at around 20 kDa that is found only in the plant extract and treatments. This plant-derived band do not seem to interact with venom components and increases its pigmentation dose-dependently. Similar bands are found in other plant extracts used as antivenoms, where there is no interaction either [63,64,65]. These preliminary findings support the existence of venom–extract interactions, although the exact mechanisms must be explored in future research.

As far as we know, this is the first insight into the chemical constituents of *U. sagittifolia.* Phytochemical screening evidenced that its ethanolic extract is rich in anti-snake metabolites, which should be protagonists in the enzymatic inhibitions reported here. Earlier studies have demonstrated the anti-inflammatory properties of phenolic compounds and the antihemorrhagic effects of flavonoids [19,66,67]. Some of them are related to the chelation of metal atoms such as Ca^2+^ and Zn^2+^, which are essential cofactors for enzymatic activity [16]. *Schizolobium parahyba*, a plant rich in flavonoids such as isoquercitrin, catechin, and allocatechin, has shown great antihemorrhagic activity against toxic activities of *B. jararacuçu* and *B. neuwiedi* venoms [61]. *Mangifera indica* extracts present high levels of phenolic compounds such as pentagalloyl, and glucopyranose has significant reduction in edema and hemorrhage in animal experimentation when compared to controls [62]. Plant extracts with a large number of alkaloids, especially aristolochic acid, ajmaline and reserpine have shown activity against envenoming [68]. Coumarins like herniarin and ayapin, isolated from *Eupatorium triplinerve* were reported to have remedies against snakebite [69]. These studies show that the main constituents of the ethanolic extract *U. sagittifolia* tubers are present in plants with antivenom activity.

Plant inhibitors are an effective and diverse alternative treatment for snakebites. To continue validating their application, researchers must investigate the extract antivenom capacities without pre-incubation, using “challenge then treat” models of envenomation in order to mimic more realistic conditions. Additionally, the synergic action of extract and traditional antivenoms of heterologous proteins should be evaluated. Purification and structural studies are necessary to assess the molecular scaffolds of metabolites present in *U. sagittifolia* extract responsible for the inhibition and neutralization of *B. atrox* venom toxins, expanding the chemical space of scaffolds to design the next generation of venom inhibitors. Furthermore, a large number of venom samples from different species is adequate to determine if the enzymatic inhibition is broad-spectrum.

## 4. Conclusions

This is the first experimental evaluation of the antivenom properties of *U. sagittifolia*, a plant involved in a long-standing tradition in rural populations along the Amazon for the management of *Bothrops* accidents. Ethanolic extracts partially protect CD-1 mice from the lethal, hemorrhagic, and inflammatory effects of *B. atrox* venom. This effect is probably due to the inhibition of toxins by abundant metabolites, such as phenolic compounds and alkaloids, which can act alone or in synergy. Future studies should purify and identify the possible bioactive compounds responsible for toxin inhibition, which constitute therapeutically useful scaffolds for the rational design of promising adjuvants tackling local effects caused by snakebites.

## 5. Materials and Methods

### 5.1. Venom Extraction

The venoms were approved for use and collection by the Ministry of Environment of Ecuador under research permits MAE-DNB-CM-2015-0017 and MAE-DNB-CM-2019-0115. A reference venom pool of *B. atrox* was obtained from specimens collected from samples of different regions of the Ecuadorian Amazon. We decided to work with a venom pool due to previously reported venomic variability [43]. To obtain dry samples, venoms were placed in 1.5 mL eppendorf tubes inside a vacuum container containing anhydrous calcium sulfate. The venom pool was stored at −35 °C until conducting in vivo and in vitro essays.

### 5.2. Plant Collection, Identification and Preparation of Ethanolic Extract

Specimens of *Urospatha sagittifolia* (Rudge) Schott were collected in the Misahuallí- Ahuano intersection road, Napo, Ecuador (1°03′21.0″ S 77°40′00.5″ W) (430 mbsl), under the Ministry of Environment permit MAAE-CMARG-0250 in July 2021. Taxonomic identification was performed by Thomas Croat at the Missouri Botanical Garden. A voucher specimen was deposited in Herbario Nacional del Ecuador (QCNE), under number QCNE-030-2021. Its tubers were washed and crushed in distilled water. The resulting material was lyophilized (Benchtop Pro 9 L, Omnitronics) for three days. The lyophilized powder was stored at −10 °C.

An aqueous extract of the lyophilized material was prepared. However, we focused on the preparation and results of the ethanolic extract since the aqueous fraction did not show an effective neutralization of the enzymatic and toxic activities of *B. atrox*. Ethanolic extract was prepared from 85 g of lyophilized tuber, which was macerated in absolute ethanol for three days in a 1:4 ratio (freeze-dried extract: ethanol). The solvent ethanol was filtered and evaporated at low pressure (100 mbar) and medium temperature (45 °C) in a Buchi Rotavapor^®^ R-300 yielding 2.7 g of extract.

### 5.3. Biochemical Composition of B. atrox Venom

We investigated the proteomic composition of the venom pool using two steps: (1) high-performance phase liquid chromatography (RP-HPLC) and (2) sodium dodecyl sulfate polyacrylamide gel electrophoresis (SDS-PAGE), according to the methodology reported by Patiño et al. (2021) [43]. In this first approach, proteins were separated, while the second analytical technique was used to identify by mass the possible class of toxins present in the sample.

For the first analysis, 1 mg of venom was dissolved in a solution of 120 μL of trifluoroacetic acid (0.1%) (Sigma-Aldrich) and 80 μL of ammonium bicarbonate (1 M) (Sigma-Aldrich) and centrifuged for 1 min to remove debris. This material was separated by reverse-phase HPLC (PREP 150 LC, Waters) equipped with the 2489 UV/Vis detector, pumps and 2545 injector, and fraction III collector. The chromatographic separation was performed on a C18 column (250 × 4.6 mm, 5 μm particle size) (SunFire Waters). The flow rate was set to 1 mL/min. The fractionation was carried out using a linear gradient of 0.1% trifluoroacetic acid (Sigma-Aldrich) (mobile phase A) and acetonitrile (≥99.9% Sigma-Aldrich) and 0.1% trifluoroacetic acid (Sigma-Aldrich) in a ratio of 66:34 (mobile phase B). Protein elution was detected at 215 nm. Each fraction was collected manually. The solvent was evaporated in a sample concentrator Genevac miVac for twelve hours at 35 °C, and samples were stored at −4 °C until electrophoresis.

Dried venom fractions obtained in the first step were submitted to electrophoresis analysis under reducing conditions. The biochemical profile was determined using a 5% concentration stacking gel and a 12.5% concentration for the running gel, in the presence of 1 M DL-Dithiothreitol (Sigma-Aldrich) at 120 mA and a temperature of 25 °C, in electrode buffer (0.025 M Tris Base (Promega), pH 8.3; 0.192 M glycine; 0.1% (m:v) SDS). Venoms solutions were prepared by 100 µg of dried fractions dissolved in 200 μL of loading buffer (0.075 M Tris-HCl, pH 6.8, 10% (v:v) glycerol, 4% (m:v) SDS, 0.001% (m:v) bromophenol blue). Molecular marker Run Blue Prestained TriColour (Blue, Red, Green); Promega was used to identify the molecular mass of venom proteins. Gels were stained with Coomassie Brilliant Blue R.

### 5.4. Enzyme Inhibition Assays

We used in vitro colorimetric assays to investigate the catalytic properties of *B. atrox* venom and its inhibition by varying ratios of the ethanolic extract of *U. sagittifolia*. The potential inhibitory was accessed in a plate reader (Glomax Discover System, Promega). For this purpose, extracts were diluted in a 1:3 (*v/v*) mixture of dimethyl sulfoxide (DMSO) and phosphate-buffered saline (PBS, pH 7.2). The extracts were prepared in a 1:12.5 (m/m), 1:25 (m/m), 1:50 (m/m) and 1:81 (m/m) ratio of ethanolic tuber powder and venom. The only extract sample contained the highest concentration of freeze-dried *U. sagittifolia* ethanolic extract used in the assay. We evaluated the enzymatic activity of the three most recognized enzymes from *B. atrox* venom: PLA_2_, SVSP and SVMP. Venom and extracts were incubated with synthetic substrates. The results were expressed as residual activity, calculated as percentages of the venom-only sample (venoms were considered as 100% activity based on enzymatic activity of positive control). Three independent experiments were carried out for each assay. Controls with the extracts at different concentrations and substrates were used to make absorbance corrections.

#### 5.4.1. Inhibition of Phospholipase A_2_

The PLA_2_ activity of *B. atrox* venom was determined according to its ability to degrade the synthetic substrate 4-nitro-3 [octanoyloxy] benzoic acid (NOBA–Sigma-Aldrich) in absence and presence of the plant extract as described by Holzer and Mackessy [58] with some modifications. The NOBA solution was prepared by dissolving 4.7 mg of this chromogenic molecule in 1.5 mL of acetonitrile (≥95% Sigma-Aldrich) and 15 mL of a buffer composed 10 mM Tris-HCl, 1 M CaCl_2_, 10 mM NaCl, pH 7.8. The venom was dissolved in Tris-HCl 50 Mm, pH 7.8 at a proportion 1:1 (m/v). Then, 220 μL of substrate solution, 20 μL of venom and 20 μL of extract at different ratios were mixed in a 96-well microplate. All the samples were incubated for 30 min at 37 °C, and the plate reader (Glomax Discover System, Promega) was set at 405 nm. The absorbance was taken immediately after the incubation and 20 min later. The enzyme activity was determined by the differences in the absorbance values in time. Absorbances obtained from venoms without incubation with the extract were defined as 100% activity.

#### 5.4.2. Inhibition of Serine Proteinases

In the case of serine proteinase, inhibition by plant extract was measured using benzoyl-arginyl-p-nitroanilide (L-BapNa) (Sigma-Aldrich) as a substrate of reference, as previously reported by Munekiyo and Mackessy [68] with some modifications. The work solution was prepared by dissolving 10.9 mg of BapNa in 250 µL of dimethylsulfoxide (DMSO–Sigma Aldrich) and 25 mL of buffer solution (10 mM Tris-HCl, 1 M CaCl_2_, 10 mM NaCl, pH 7.8). The venom was dissolved in Tris-Hcl 50 Mm, pH 7.8 at a concentration of 1 mg/mL. To measure the activity, 20 μL of venom was mixed with 200 μL of substrate. Then, 25 μL of buffer A was added to the previous solution with 25 μL of extract in a 96-well microplate. These samples were incubated for 30 min at 37 °C. The plate reader was set at an absorbance of 405 nm. The absorbance was taken immediately after the incubation and 30 min later. The enzyme activity was determined by time differences in absorbance values.

#### 5.4.3. Inhibition of Caseinolytic Activity

Azocasein (Sigma-Aldrich) was used as a substrate to determine the possible inhibition of caseinolytic activity by ethanolic extract, similar to protocol described by Munekiyo and Mackessy [70]. A solution was prepared with 40 mg of azocasein diluted in 8 mL of Tris-HCl 50 Mm, pH 7.8 buffer solution. The *B. atrox* venom was dissolved in the same buffer with a concentration of 1 mg/mL. Ten µL of venom was mixed with 90 µL of substrate solution and 20 µL of extract for inhibition determination. The solution was incubated for 90 min at 37 °C and then 200 μL of 5% TCA (≥99.0% Sigma-Aldrich) was added to all samples and centrifuged at 8000 rpm for 5 min. Later, 150 μL of the supernatant was taken and placed in a 96-well microplate and mixed with 150 μL of 0.5 M NaOH (Merk). To measure absorbance, the plate reader (Glomax Discover System, Promega) was set at 450 nm. Enzyme activity was determined by comparing corrected absorbance values.

### 5.5. In Vivo Experimentation

Male CD-1 mice (20–22 g) were provided by the Instituto Nacional de Investigación en Salud Pública Dr. Leopoldo Izquieta Pérez (Guayaquil, Ecuador). The mice received food and water ad libitum. They were housed in cages maintained under standard conditions (25 °C, 12 h light/dark cycles), and their health was monitored daily during acclimatization. All experiments were conducted in accordance with the European Union recommendations on animal testing (European Council Directive 2010/63/EU). World Health Organization recommendations for research related to preclinical antivenom evaluation were also taken into account. We adhered to protocols approved by the Research Coordination of the Universidad Regional Amazónica Ikiam. All the procedures complied with the 3R-based design for animal experimentation [71].

#### 5.5.1. Survival Curve

We used 2× the intraperitoneal LD50 doses of *B. atrox* venoms, based on studies previously published by our research group. Overall, groups of four mice injected intraperitoneally with venom and different venom:extract ratios (1:12.5/1:25/1:50/1:81) preincubated for 30 min at 37 °C. Controls with vehicle and only plant extract (highest concentration of freeze-dried *U. sagittifolia* ethanolic extract used in the assay) were also evaluated. Behavioral changes and mortality were recorded for 48 h; surviving mice were euthanized by cervical dislocation at the end of the experiment.

#### 5.5.2. Edema

The mice were randomly divided into groups (*n* = 4). Edema was induced by the subcutaneous injection of *B. atrox* venom (10 µg) into the plantar surface of the left hind paw of each animal. Then, the venom with the extracts at different ratios and controls (the same mentioned above), previously incubated at 37 °C and 30 min, was injected into the plantar surface of the left hind leg. Vehicle and extract alone were also evaluated. All samples had a final volume of 50 µL. Paw diameter was measured before venom injection and 30 min later, using a vernier caliper. Edema was expressed as the increase in paw diameter (mm) after venom injection relative to the pre-injection value for each animal.

#### 5.5.3. Hemorrhage

Animals were randomly divided into different groups (*n* = 4). One group received only 15 μg of venom in the dorsal area. Other groups, received an injection of 15 μg of venom with different ratios of extract incubated for 30 min at 37 °C. The control groups received vehicle and crude extract under the same conditions. After three hours, the mice were euthanized by cervical dislocation to dissect the dorsal skin and measure the hemorrhage area (cm2). Photographs of the red halos were taken and then analyzed with ImageJ.

### 5.6. Venom–Extract Interaction by SDS-PAGE

To determine if the different ratios of the extract degrade, precipitate, form molecular complexes and interact with venom proteins, an SDS-PAGE was performed with the same conditions described in Biochemical composition of *B. atrox* venom. For this analysis, 10 μL of venom and 10 μL of plant extracts were used at different ratios and preincubated at 37 °C for 30 min before SDS-PAGE running. The band intensities were analyzed with ImageJ software from digital images of the gel.

### 5.7. Insights into Phytochemical Content of U. sagittifolia Extract

The phytochemical profile of the ethanolic extract from *U. sagittifolia* was investigated using qualitative and quantitative approaches. Initially, the presence of a wide range of metabolites including alkaloids, saponins, terpenoids, tannins, quinones, coumarins, phenolic, flavonoids, and anthocyanins were detected [72,73]. The qualitative analyses were classified as strongly positive (+++), positive (++), weakly positive (+), and non-detected (-) according to Caro et al. (2019) [20].

In the second strategy, we measured the content of phenolic compounds and flavonoids due to the recognized antivenom potential of this metabolites. The first group was quantified using the Fólin–Ciocalteu method [74,75]. For this purpose, gallic acid (GA) was used as a standard. The sample was mixed with the Folin reagent (0.1 M) protecting it from light. After 5 min Na_2_CO_3_ (7.5%) was added and leave to stand for 120 min. The absorbance was measured at 755 nm and the results were expressed as mg of gallic acid per gram of *U. sagittifolia* extract [76]. On the other hand, flavonoids were determined by the aluminum trichloride protocol with some modifications [76] The samples were mixed with Na_2_NO_3_ (5%) and leave to stand for 6 min. Then, AlCl_3_ (10%) was added leaving to stand for 5 min. In the last step, NaOH (1 M) was added, and the absorbance was measured at 510 nm after 15 min. Results are presented as mg of catechin (CT) per gram of *U. sagittifolia* extract [77]. Furthermore, the procedures validated for standards and samples showed a linearity of R^2^ > 0.9987 with a linear regression y = 1.985 x + 0.236 using concentrations between 2 mg/L to 10 mg/L of GA to determinate polyphenols. On other hand, the linearity was R^2^ > 0.9889 with a linear regression y = 0.035 x − 0.004 using concentrations between 0.8 mg/L and 4.0 mg/L of CT to estimate the concentration of flavonoids. The experimental limit of detection and limit of quantification were between 0.0269 mg/L and 0.0356 mg/L to GA and 0.430 mg/L and 0.620 mg/L to CT, respectively.

### 5.8. Statistical Analyses

We used the t tests for two independent samples to verify if there were significant differences when comparing the results in toxic and enzymatic effects obtained between the venom alone and the samples with different ratios of extract. The level of significance was established at *p* ≤ 0.05. Analyses and graphs were produced in OriginLab.

## Figures and Tables

**Table 1:** Phytochemical screening of ethanolic extract from *Urospatha sagittifolia*. Presence (+) and absence (-) of secondary metabolites in *U. sagittifolia*; number of signals is related to the intensity of reaction.

Specialized Metabolite	Ethanolic Extract
Alkaloids	+++
Saponins	++
Terpenoids	+
Tannins	++
Quinones	-
Coumarins	+++
Phenolic compounds	+++
Flavonoids	+++
Anthocyanins	-

**Table 2:** Plants as antivenom agents. Various methods of extraction, parts of plants and specialized metabolites have been tested with different species of venomous snakes.

Plant	Extraction/Metabolite	Inhibitory Effects on	Snake Venom	Reference
*Aristolochia indica*	Aqueous extract of the roots	L-amino acid oxidase and protease	*Daboia russelii*	[32]
*Azadirachta indica*	Purified molecule (AIPLAI) from the methanolic leaf extract	PLA_2_ activity	*Naja naja*, *Naja kaouthia* and *Daboia russelli*	[33]
*Philodendron megalophyllum*	Aqueous extract using the stem	Hemorrhagic and fibrinolytic activities	*B. atrox*	[19]
*Citrus limon, Citrus sinensis, Citrus aurantium*	Hesperetinb obtained from hesperidin in citrus peels	Serine proteinase activity	*Crotalus simus*	[45]
*Pentaclethra macroloba*	Purified molecules of the aqueous extract of the bark. (macrolobin-A and B)	Hemorrhagic, proteolytic, and fibrinolytic activities	*B. neuwiedi* and *B. jararacussu*	[46]
*Dracontium dubium*	Ethanolic extract from tubers	Lethality, inflammatory, and hemolytic effects	*B. asper*	[20]
*Piper longum*	Ethanolic extract of the fruits	Lethality, hemorrhage, necrosis, defibrinogenation, and inflammatory paw edema	*Daboia russelli*	[47]
*Cordia verbenacea*	Methanolic extracts of leaves	Paw edema	*B. jararacussu*	[48]
*Urera bacífera,* *Loasa speciosa* *Chaptalia nutans* *Satureja viminea*	Aqueous extracts of leaves	Edema	*B. asper*	[49]
*Uncaria tomentosa*	Aqueous extracts of roots	Edema	*B. asper*	[49]
*Mouriri pusa, Davilla elliptica, Byrsonima crassa*	Methanolic extracts from leaves	Hemorrhage	*B. jararaca*	[50]
*Bursera* *Simaruba, Phoebe* *Brenesii, Virola koschnyi*	Extraction water:ethanol (85:15) of barks	Hemorrhage	*B. asper*	[52]
*Clusia palmana, Croton* *Draco*	Extraction water:ethanol (85:15) of leaves	Hemorrhage	*B. asper*	[52]
*Persea* *americana*	Extraction water:ethanol (85:15) of seed	Hemorrhage	*B. asper*	[52]
*Harpalyce brasiliana*	Purified edunol extracted from roots	PLA_2_ activity	*B. jararacucu*	[13]
*Brongniartia podalyrioides*	Purified edunol isolated from roots	Lethality	*B. atrox*	[60]
*Schizolobium parahyba*	Purificated molecules from aqueous extract of leaves (isoquercitrin, myricetin-3-O-glucoside, catechin, and gallocatechin)	Hemorrhagic and fibrinogenolytic activities	*B. alternatus*	[61]
*Mangifera indica*	Ethanolic extract from seeds	Caseinolytic and fibrinogenolytic activities	*Calloselasma rhodostoma* *Naja siamensis*	[62]

## Data Availability

The original contributions presented in this manuscript are publicly available.
